# In Silico Analysis of Missense Mutations as a First Step in Functional Studies: Examples from Two Sphingolipidoses

**DOI:** 10.3390/ijms19113409

**Published:** 2018-10-31

**Authors:** Ana Joana Duarte, Diogo Ribeiro, Luciana Moreira, Olga Amaral

**Affiliations:** 1 Instituto Nacional de Saúde Dr Ricardo Jorge (INSA, IP), Departamento de Genética Humana, Unidade de Investigação e Desenvolvimento, Rua Alexandre Herculano 321, 4000-055 Porto, Portugal; ana.duarte@insa.min-saude.pt (A.J.D.); diogo.ribeiro@insa.min-saude.pt (D.R.); luciana.moreira@insa.min-saude.pt (L.M.); 2 ICBAS, University of Porto, 4099-002 Porto, Portugal; 3 CECA, ICETA, University of Porto, 4099-002 Porto, Portugal

**Keywords:** lysosomal glucocerebrosidase, lysosomal alpha-galactosidase, sphingolipidoses, functional studies, in silico analysis, *GBA1*, *GLA*, *CSTB*, *ARSA*, *GALC*

## Abstract

In order to delineate a better approach to functional studies, we have selected 23 missense mutations distributed in different domains of two lysosomal enzymes, to be studied by in silico analysis. In silico analysis of mutations relies on computational modeling to predict their effects. Various computational platforms are currently available to check the probable causality of mutations encountered in patients at the protein and at the RNA levels. In this work we used four different platforms freely available online (Protein Variation Effect Analyzer- PROVEAN, PolyPhen-2, Swiss-model Expert Protein Analysis System—ExPASy, and SNAP2) to check amino acid substitutions and their effect at the protein level. The existence of functional studies, regarding the amino acid substitutions, led to the selection of the distinct protein mutants. Functional data were used to compare the results obtained with different bioinformatics tools. With the advent of next-generation sequencing, it is not feasible to carry out functional tests in all the variants detected. In silico analysis seems to be useful for the delineation of which mutants are worth studying through functional studies. Therefore, prediction of the mutation impact at the protein level, applying computational analysis, confers the means to rapidly provide a prognosis value to genotyping results, making it potentially valuable for patient care as well as research purposes. The present work points to the need to carry out functional studies in mutations that might look neutral. Moreover, it should be noted that single nucleotide polymorphisms (SNPs), occurring in coding and non-coding regions, may lead to RNA alterations and should be systematically verified. Functional studies can gain from a preliminary multi-step approach, such as the one proposed here.

## 1. Introduction

Lysosomal storage diseases (LSDs) are a large group of inherited disorders leading to various clinical symptoms, caused by defects in lysosomal enzymes, transporter proteins, activator proteins, or other proteins involved in lysosomal function or biogenesis. Such defects lead to total or partial loss of enzyme activity and consequent accumulation of substrate, which results in impaired organelle function, leading to subsequent multi-organ dysfunction. The enzymes involved in two of the less rare LSDs are lysosomal glucocerebrosidase (GlcCerase, glucosylceramidase or acid-β-glucosidase, EC 3.2.1.45), and lysosomal acid-α-galactosidase (α-GAL or α-Gal A, EC 3.2.1.22). Most commonly, mutations in the genes coding for the aforementioned enzymes, *GBA1* (ID: 2629) and *GLA* (ID: 2717) lead to the development of Gaucher [1] and Fabry diseases, respectively [2]. Gaucher disease (GD, MIM #230800, 230900, 231000) is the most common lysosomal storage disorder [1], and Fabry disease (FD, MIM #301500) is an X-linked disorder that has a large range of phenotypes [2]. In the case of both shingolipidoses, therapeutic approaches based on enzyme replacement or small-molecule compounds have been successfully developed. Interestingly, both hydrolytic reactions require activation by saposins; whereas globotriaosylceramide binds saposin B (SapB) prior to presentation to α-GAL, saposin C (SapC) enhances GlcCerase activity [3]. The enzyme GlcCerase is a peripheral membrane protein that catalyzes the hydrolysis of glucosylceramide (GlcCer) to ceramide and glucose. The three-dimensional (3D) structure of human GlcCerase was discovered by Dvir et al. in 2003; it comprises three non-contiguous domains, with the catalytic site located on the (α/β)_8_ TIM barrel in domain III [4]. The 3D structure of human α-GAL was found by Garman and Garboczi in 2004; it is composed of two domains and shares with GlcCerase a (α/β)_8_ TIM barrel motif in the catalytic domain and an immunoglobulin fold in the same relative position [5].

To date, hundreds of mutations in *GBA1* and *GLA* genes have been identified (http://www.hgmd.cf.ac.uk), and new sequencing techniques continuously identify variants in large numbers (http://gnomad.broadinstitute.org/). Large sets of information were also recently assembled through the 1000 genomes project (http://www.internationalgenome.org/1000-genomes-browsers/).

The term in silico goes back to the 1970s and is related to the computer component silicon. In silico methods are based on computational approaches for the prediction of effects prior to development of laboratory methods [6] In vitro functional studies involve laboratory assays for testing various types of functions and usually rely on cell based overexpression systems and cell based “test-tube” assays. The in vitro studies require many consumables, specific laboratory apparatus and time-consuming optimizations and laboratory procedures. Computational modeling is an important tool to deal with the rapid increase in bioinformatics information. Presently, the most rapid and inexpensive way to predict whether a Single-Nucleotide Polymorphism (SNP) will potentially cause disease is by performing a computational analysis. Different parameters, such as clinical, populational, structural, and bioinformatics, need to be considered when analyzing results. The increasing number of computational tools available, and the rapidly growing number of available crystal structures, has turned in silico modeling into an accurate complementary, and often crucial, prediction methodology. Different computational platforms take into account, to various degrees, factors such as the general rules of protein chemistry, 3D structure, and homologies in amino acid sequences among various species or related proteins [7]. Most of the *GBA1* and *GLA* mutations are point mutations leading to missense substitutions, and functional studies exist in several of those mutations. The existence of in vitro data adds relevant information for the computational study of the molecular basis of the protein impairment.

In this report 23 single nucleotide alterations in the *GBA1* and *GLA* genes, leading to amino acid substitutions with protein functional studies, were re-examined through in silico analysis and their effects were predicted and compared using different platforms available online. The analyzed amino acid substitutions were individually mapped into the available 3D enzyme model, using the PyMOL tool [8]. Comparison of the computational data obtained with previous in vitro expression, or functional, data was carried out to evaluate the prediction accuracy regarding the establishment of genotype/phenotype correlation. In silico prediction of the amino acid substitution impact at the protein level may, sometimes, be considered as an alternative to in vitro expression or as a pre-study indicator of the need for research at the functional level. Attentive in silico analysis is a potentially valuable option for immediate guidance regarding patient care and counseling, being able to confer an indication of prognosis value to the genotyping results.

Functional effects of mutations were predicted with different tools in an attempt to distinguish between variant amino acid substitutions, considering evolutionary information, structural features, and other relevant information. Tools that combine different types of existing information are more complete and were used in the present study [9,10,11]. Missense mutations were selected in different domains of the GlcCerase and of the α-GAL proteins with different types of functional evidence of causality. In order to broaden the scope of the study, mutations in three other genes related to neurodegenerative diseases were also added to the present study.

## 2. Results

The aim of this work was to investigate the prediction value of different bioinformatics tools, applying them to single amino acid substitutions in the *GBA1* and *GLA* genes.

**Figure 1:**
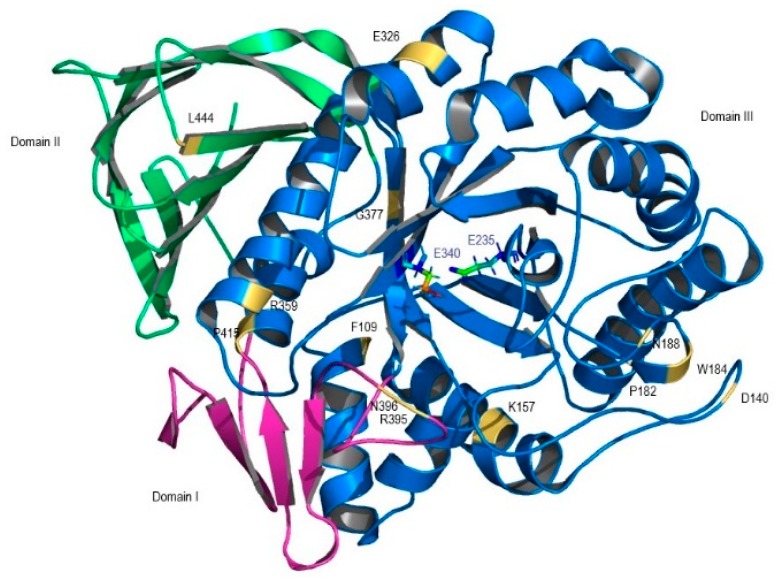
Location of GlcCerase missense mutations considered in this study presented in a solid ribbon model. Domain I is shown in pink, domain II is in green, and domain III is shown is blue. Mutations are identified in yellow. Active site residues (E235 and E340, in blue letters) are shown as ball-and-stick model, with different colors.

**Figure 2:**
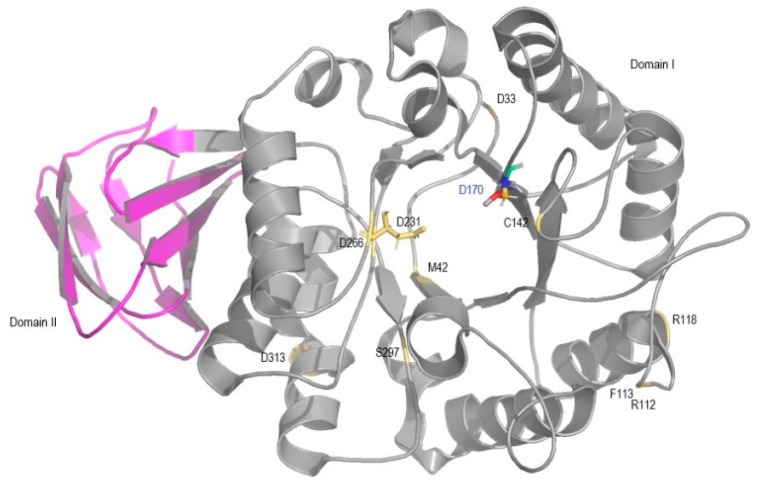
Location of α-GAL missense mutations considered in this study presented in a solid ribbon model. Domain I is shown in grey and domain II is shown in pink. Mutations are identified in yellow, and active site residues (D170 and D231, the non-mutated active site residue in blue letters) are shown as ball-and-stick model, with different colors.

GlcCerase and α-GAL structures were obtained from the Protein Data Bank (PDB). *GBA1* mutations (p.F109V, p.P182L, p.D140H, p.K157Q, p.W184R, p.N188S, p.E326K, p.R359Q, p.G377S, p.R395P, p.N396T, p.P415R, and p.L444P) and *GLA* mutations (p.D33G, p.M42V, p.R112C, p.F113L, p.R118C, p.C142W, p.D231G, p.D266N, p.S297F and p.D313Y) were mapped into 3D GlcCerase and α-GAL structures: the first X-ray human GlcCerase to be solved (PDB code 1OGS) [4] and into the first 3D α-GAL structure (PDB code 1R46) [5] (Figure 1 and Figure 2). Three-dimensional structures were designed using PyMOL (http://www.pymol.org) in order to visualize how these alterations could affect enzyme structure.

The missense mutations, depicted in Figure 1 and Figure 2, were computationally analyzed and the retrieved results were compared with the in vitro results and functional data (Table 1), in order to ascertain the validity of the platforms used and evaluate the prediction accuracy regarding the establishment of genotype/phenotype correlation. Differences in the results obtained reflect the different types of algorithms used in the computational platforms.

In order to broaden the scope of the present study, we analyzed 14 additional mutations in other genes involved in neurodegenerative lysosomal-related disorders (Table 2). In all cases, functional studies were available. These previous mutations provided an ampler comparison between in vitro and in silico results.

## 3. Discussion

In vitro mutagenesis and subsequent expression of mutant proteins, or functional studies and characterization, is a cumbersome task in terms of time, workload, and cost. For these reasons, in silico analysis is a desirable, fast, inexpensive, and reliable way to boost our understanding of how an amino acid substitution could affect the protein structure and function. Availability of 3D protein structures enables the mapping of amino acid substitutions and, therefore, helps complement the information acquired from different computational platforms. These aspects facilitate preliminary research in the biomedical field. As observed with the tools used here, the incorporation of more data increases the accuracy of the results, and thus makes predictions more reliable.

When a novel missense mutation is detected in a disease context, and its polymorphic nature has been excluded by population studies, it is possible to predict its outcome through in silico analyses, by first performing computational SNP evaluation followed by modeling the amino acid substitution into the 3D protein structure. In silico analysis is necessary to predict the impact of novel mutations in diseases such as the lysosomal disorders analyzed here. However, general limitations exist—for instance, the structural-based prediction tools may be unable to accurately predict mutation effects due to a lack of homologous structures in the databases. In such cases, functional analysis studies should be performed to elucidate how the missense mutation affects the protein function and contributes to the patient phenotype.

Overall, the retrieved results from the different computational platforms were rather similar, although they use different data sources and algorithms. The biggest difference observed seemed to be between PROVEAN and the other platforms, since it takes into account fewer variables. On the other hand, SNAP2 relies on protein and DNA data, as well as evolutionary and conservation information, and therefore is able to check more aspects regarding the impact of amino acid substitutions. In the case of α-GAL and GlcCerase mutations p.F113L and p.W184R, the location on the periphery of the proteins could suggest that they did not have a significant effect on enzyme activity and stability. However, the computational studies indicate them as damaging missense mutations and in vitro studies confirm that the respective proteins are unstable with reduced activity (p.F113L) or even inactive (p.W184R) [12,19]. These types of mutations usually occur in specific protein binding sites. These specific amino acids can be located on sites that are vital for the dimerization in α-GAL or tetramer formation in GlcCerase [38], or be located in sites where the activator proteins (Saposin B in α-GAL and Saposin C in GlcCerase) binds. Binding disruption will lead to partial or total loss of protein function.

A major limitation of this study is that there are few neutral, or low-score, variants to be analyzed. This problem arises because studies are not exhaustive enough and mutations that may look neutral are often not sufficiently investigated. Particular attention should be given to mutations in the “milder” spectrum. In addition to amino acid substitutions, SNPs may alter RNA processing by interfering with consensus sequences. A silent mutation or a “neutral” amino acid substitution may alter consensus sequences involved in splicing and lead to abnormal transcripts. Such mutations risk being overlooked and labeled as non-causal. An example to take into account is that of an apparently neutral/silent mutation on the *CSTB* gene (p.Q22Q in Table 2), which affected RNA processing and was proven to be causal only by functional studies [39,40].

Limitations of in silico analysis also arise since mutations (in the patient) may have additive or compensatory effects and the tools used only predict single protein changes. Besides the wide range of mutant variants and clinical phenotypes, in some cases, mutations in the same gene may be associated with more than one disease. Certain *GBA1* mutations are known to be associated with Gaucher Disease (GD) and with Parkinson’s disease (PD) [41,42]. A good example of this association is mutation p.E326K, which has been repeatedly investigated [14,38,43]. The association of a single protein with different diseases is an additional limitation for in vivo and in vitro assays. Recently, a complex integration of in silico computational analysis has been used for the understanding of the association of *GBA1* mutations in GD and PD [44]. This latter approach, integrating multiple parameters, namely molecular dynamics, seems to pave the way for the development of more dependable in silico computational modeling approaches.

In general, it is possible to conclude that in silico methods remain an accurate way to make a rapid analysis regarding the expected effect of mutations. Nonetheless, the more factors that are taken into account, the more accurate the prediction will be. In order to take the best advantage of in silico analysis, different computational platforms should be used, trying to cover the major factors influencing protein structure and function. RNA processing alterations should also be routinely investigated by in silico analysis. The SNP impact at the RNA level can be investigated by using some of the various RNA assessment tools, such as Human Splicing Finder [45], GeneSplicer [46], NetGene2 [47], or Berkeley Drosophila Genome Project (BDGP) Splice Prediction by Neural Network [48].

## 4. Materials and Methods

### 4.1. Data Identifiers

The UniProt database was used to retrieve information about the protein sequence of *GBA* (UniProtKB P04062), *GLA* (UniProtKB P06280), *CSTB* (UniProtKB P04080), *ARSA* (UniProtKB P15289) and *GALC* (UniProtKB P54803). The three-dimensional structure of these proteins was obtained from the Protein Data Bank (PDB, http://www.rcsb.org/pdb/home/home.do) with the following 3D reference IDs: GlcCerase (1OGS), α-GAL (1R46), Cystatin-B (2OCT) for CSTB gene, Arylsulfatase A (1AUK) for ARSA gene, and Galactocerebrosidase (3ZR5) for GALC gene.

### 4.2. In Silico Methods

Twenty-three missense mutations were analyzed using four different computational platforms freely available online (Table 1). PROVEAN (Protein Variation Effect Analyzer) (http://provean.jcvi.org/) is a computational tool that predicts whether an amino acid substitution or indel will have an impact on the biological function of a protein. PROVEAN is useful for filtering sequence variants to identify nonsynonymous or indel variants that are predicted to be functionally important. Results are given as “deleterious” or “neutral”, according to scores [49,50]. The PolyPhen-2 (Polymorphism Phenotyping v2) program (http://genetics.bwh.harvard.edu/pph2/) uses the sequence homology and knowledge of 3D structures; it predicts the possible impact of an amino acid substitution on the structure and function of a human protein using straightforward physical and comparative considerations. The results are classified as “benign”, “possibly damaging”, “probably damaging”, or “unknown” [7,51]. The ExPASy Swiss-model [52] is a fully automated protein structure modeling server, accessible via the ExPASy web page (https://swissmodel.expasy.org/), and was also used in this study [53]. The SNAP2 (screening for non-acceptable polymorphisms) program (www.rostlab.org/services/SNAP/) incorporates evolutionary information, predicted aspects of protein structure, and other relevant information in order to make predictions regarding the functionality of mutated proteins [11]. The results are retrieved as “having an effect” or “being neutral”, and a score, correlated with the severity of the change, is given for each substitution along with the percentage of expected accuracy [10].

## 5. Conclusions

In the present work, we show that a comparison of the results between various platforms is crucial and, in the case of the most deleterious mutants, the results are generally clear. In the case of the more neutral mutations, functional studies and more refined in silico approaches are fundamental for the understanding of the mutation’s impact on the RNA processing, protein function, and pathophysiology of the disease.

## Figures and Tables

**Table 1:** In silico analysis comparison of 23 missense mutations in the GBA1 and GLA genes.

Gene Mutants	PROVEAN	PolyPhen-2	SNAP2	ExPASy	Protein Function and Structure
			*Prediction Expected Accuracy*		
*GBA1* Mutants					
**F109V** **CM005404**	Deleterious	Probably damaging	Effect 75%	NA	15% of wt activity; weakly conserved; domain III; stable protein [12]
**P182L** **rs80205046**	Deleterious	Probably damaging	Effect 85%	NA	Near null activity; buried site; domain III; unstable protein [13]
**D140H** **rs147138516**	Neutral	Benign	Neutral 57%	Disease	73% of wt activity; domain III [14]
**K157Q** **rs121908297**	Deleterious	Probably damaging	Neutral 57%	Disease	9.7% of wt activity; domain III; conserved region; unstable protein [14]
**W184R** **rs61748906**	Deleterious	Probably Damaging	Effect 66%	Disease	Inactive enzyme; domain III periphery; alteration of enzyme geometry; unstable protein [12]
**N188S** **rs364897**	Deleterious	Benign	Effect 66%	Disease	66.6% of wt activity; domain III periphery; stable protein [13]
**E326K** **rs2230288**	Neutral	Benign	Neutral 56%	Disease	42.7–25% of wt activity; domain III; stable protein [15]
**R359Q** **rs74979486**	Deleterious	Probably Damaging	Effect 75%	Disease	4.5% of wt activity; domain III stable protein; highly conserved region [12]
**G377S** **rs121908311**	Deleterious	Probably Damaging	Effect 91%	Disease	17% of wt activity; domain III; stable protein [12]
**R395P**	Deleterious	Benign	Effect 75%	NA	4.5% of wt activity; domain I, loop 2; stable protein [12]
**N396T** **rs75385858**	Deleterious	Probably Damaging	Effect 85%	NA	14% of wt activity; domain I; stable protein [12]
**P415R** **rs121908295**	Deleterious	Probably damaging	Effect 59%	Disease	Near null activity; conserved region; unstable protein [16]
**L444P** **rs421016**	Deleterious	Possibly damaging	Effect 91%	NA	5.7–9% of wt activity; unstable protein [12,17].
***GLA* Mutants**					
**D33G** **rs869312136**	Deleterious	Possibly damaging	Effect 75%	Unclassified	37% of wt activity; periphery of domain I [18]
**M42V**	Deleterious	Probably damaging	Effect 85%	Disease	7% of wt activity; domain I; unstable protein [19]
**R112C** **rs104894834**	Deleterious	Probably damaging	Effect 91%	Disease	5% of wt activity; periphery of domain I; unstable protein [19]
**F113L** **rs869312142**	Deleterious	Probably damaging	Effect 91%	Disease	20% of wt activity; periphery of domain I; altered alpha-GAL surface; unstable protein [19]
**R118C** **rs148158093**	Deleterious	Probably Damaging	Effect 53%	NA	29–32% of wt activity; periphery of domain I; unstable protein [20,21].
**C142W**	Deleterious	Probably damaging	Effect 95%	NA	5% of wt activity; domain I; near active site pocket; unstable protein [19].
**D231G**	Deleterious	Probably damaging	Effect 95%	NA	4% of wt activity; domain I, active site pocket; stable protein [19]
**D266N** **rs869312407**	Deleterious	Probably damaging	Effect 95%	Disease	5% of wt activity; domain I, near the active site pocket; buried; unstable protein [19]
**S297F** **rs28935489**	Deleterious	Probably damaging	Effect 95%	Disease	5% of wt activity; unstable protein [19]
**D313Y** **rs28935490**	Deleterious	Probably damaging	Effect 95%	Disease	76% of wt activity; in domain I periphery; stable protein [22,23]

Legend: wt—wild-type; NA—Results not available with that computational tool.

**Table 2:** In silico analysis of 14 other single nucleotide mutations in the genes *ARSA* (MIM ID 607574) and *GALC* (MIM ID 606890), *CSTB* (MIM ID 601145).

Gene Mutants	PROVEAN	PolyPhen-2	SNAP2	ExPASy	Protein Function and Structure
			*Prediction Expected Accuracy*		
*ARSA* mutants					
**G86D** **rs74315460**	Deleterious	Probably damaging	Effect 95%	Disease	Null activity; unstable protein [24]
**C156R** **rs199476348**	Deleterious	Probably Damaging	Effect 59%	Disease	50% of wt activity [25]
**T274M** **rs74315472**	Deleterious	Probably Damaging	Effect 95%	Disease	35% of wt activity [26]
**C300F** **rs74315484**	Deleterious	Probably Damaging	Effect 95%	Disease	Null activity; disruption of disulfide bond linking major and minor β-sheets [27,28]
**T409I** **rs74315481**	Neutral	Possibly damaging	Effect 75%	Disease	60% of wt activity [29]
***GALC* Mutants**					
**I82M** **without reference SNP (rs)**	Deleterious	Probably Damaging	Neutral 57%	Disease	Normal activity [30]
**G286D** **rs199847983**	Deleterious	Probably Damaging	Effect 71%	Disease	17.5% of wt activity [31]
**Y335C** **rs757407613**	Deleterious	Probably Damaging	Effect 75%	Disease	10% of wt activity [32]
**G553R** **rs748573754**	Deleterious	Probably Damaging	Effect 91%	Disease	1.8% of wt activity [31]
**L634S** **rs138577661**	Deleterious	Probably Damaging	Effect 95%	Disease	12% of wt activity [30]
***CSTB* mutants**					
**Q22Q** **rs386833443**	Neutral	NA	Neutral 82%	NA	Expected abnormal peptide with premature truncation [33]
**G4R** **rs74315443**	Deleterious	Probably Damaging	Effect 85%	Disease	Binding pocket modification; interaction properties compromised [34]
**G50E** **rs312262708**	Deleterious	Possibly Damaging	Effect 95%	NA	Altered stability and interaction with target proteins [35,36]
**Q71P** **rs796052392**	Deleterious	Possibly Damaging	Effect 75%	NA	Changes in second binding loop; altered binding affinities [37]

Legend: wt—wild-type; NA—Results not available with that computational tool.
